# Artificial Intelligence in Patient-Centered Care and Macro-, Meso-, and Micro-Level Determinants of Rehumanization and Dehumanization: Qualitative Interview Study

**DOI:** 10.2196/82774

**Published:** 2026-05-27

**Authors:** Dora Horvath, Noemi Szilvia Lorincz

**Affiliations:** 1 Department of Strategic Management, Corvinus University of Budapest, Fovam Square 8, Budapest, 1093, Hungary, +36 1 482 5000

**Keywords:** artificial intelligence, AI integration, qualitative research, digital transformation, patient-centered care

## Abstract

**Background:**

Patient-centered care remains a foundational principle of modern health care. The digital transformation of health systems has accelerated the adoption of artificial intelligence (AI) across diagnostic, predictive, and communicative functions, with implications for efficiency and clinical workflows. At the same time, AI integration raises concerns regarding transparency, equity, accountability, and trust, positioning it as a potential driver of both rehumanizing and dehumanizing dynamics in health care practice.

**Objective:**

This study examines how the adoption of AI in health care may influence patient-centered care, exploring its potential to promote rehumanization or contribute to dehumanization. The objective is to identify the factors that shape these outcomes at the macrolevel (policy and infrastructure), mesolevel (institutional practices), and microlevel (individual behaviors and interactions).

**Methods:**

This study adopts an exploratory qualitative design informed by grounded theory principles, drawing on 20 semistructured interviews with health care leaders, clinicians, researchers, legal experts, and industry consultants who have substantial professional experience across European health care systems, with some participants also contributing experience from the US health care context. To enhance analytical rigor and transparency, the study applied the Gioia methodology, enabling inductive coding from first-order concepts to second-order themes and aggregate dimensions. This multistakeholder approach facilitated a nuanced examination of how AI integration is perceived and experienced across macro-, meso-, and microlevels of health care.

**Results:**

The analysis identified key system-level factors shaping rehumanizing or dehumanizing outcomes of AI integration. At the macrolevel, 8 factors—including regulatory frameworks, policy priorities, and infrastructure—were identified as influencing whether efficiency pressures outweigh patient-centered values. At the mesolevel, 5 factors related to institutional strategies, workflows, and leadership shape how AI tools are embedded into care delivery. At the microlevel, 7 factors related to individual behaviors, trust, and doctor-patient interaction dynamics influence whether AI supports empathy and engagement or diminishes them. Rehumanizing potentials include reduced administrative burden, improved care pathways, clearer health communication, and enhanced decision-making, while risks include shorter consultations, reduced empathy, overreliance on automation, and erosion of professional identity. Without deliberate alignment with patient-centered principles, efficiency gains risk undermining the human dimensions of care.

**Conclusions:**

This study represents one of the first empirical examinations of how AI shapes health care practices through rehumanizing and dehumanizing dynamics. The findings demonstrate that outcomes depend not only on technical capabilities but also on regulatory frameworks, institutional strategies, and cultural adaptation. By systematically mapping influencing factors across macro-, meso-, and microlevels, the research provides actionable insights for decision-makers to ensure that efficiency gains remain aligned with patient-centered principles. Realizing AI’s promise requires coordinated action to preserve empathy, trust, and interpersonal connection, ensuring that innovation strengthens rather than weakens the human dimensions of care.

## Introduction

### AI and the Human Dimension of Patient-Centered Care

Patient-centered care, grounded in respect, empathy, effective communication, and shared decision-making, remains a cornerstone of modern health care [1]. At its core, patient-centered care reflects a broader effort to humanize care by recognizing patients as whole persons rather than as diagnoses or data points [2,3]. At the same time, health care systems are undergoing rapid digital transformation, with artificial intelligence (AI) emerging as a central driver of change. AI applications in clinical decision support, diagnostics, predictive analytics, personalized treatment planning, and patient communication promise improvements in accuracy, efficiency, and resource allocation [4,5]. By reducing administrative burdens and supporting clinical reasoning, AI may indirectly strengthen patient-centered care by enabling clinicians to devote more time to meaningful patient engagement [6].

However, the expanding role of AI has also intensified concerns about potential dehumanization in health care. Humanization refers to care that acknowledges patients’ lived experiences, dignity, and agency, whereas dehumanization involves processes—often unintended—through which individuals are reduced to objects, categories, or data [7,8]. Although AI holds significant promise, its integration raises critical ethical and organizational challenges, including algorithmic bias, limited transparency, data quality concerns, regulatory uncertainty, and shifting accountability structures [9]. If inadequately addressed, these challenges may alter professional roles and patient-provider relationships in ways that risk weakening empathy, trust, and relational continuity [10].

AI integration thus represents a pivotal juncture: it may contribute either to rehumanization—by enhancing communication, personalization, and access—or to dehumanization, particularly if it replaces rather than supports interpersonal interaction [11-14]. Importantly, these trajectories are shaped not only by technological capabilities but also by contextual conditions at the macrolevel (policy and regulatory), mesolevel (organizational), and microlevel (individual and relational) [15]. While conceptual debates on AI and human-centered care are growing, empirical research examining how these multilevel factors interact in practice remains limited.

To address this gap, this study explores how AI implementation in health care shapes processes of rehumanization and dehumanization, and which macro-, meso-, and microlevel factors influence these dynamics. Drawing on 20 semistructured interviews with experts across the health care sector and applying an inductive qualitative approach using the Gioia methodology, the study provides a multilevel analysis of how AI is perceived, interpreted, and experienced in relation to the human dimension of care. By integrating perspectives from policy, organizational, and clinical contexts, it aims to generate empirically grounded insights into the conditions under which AI may reinforce—or undermine—patient-centered health care.

### Literature Background: Rehumanization and Dehumanization in Health Care

Using the framework established by Todres et al [2], the humanization of health care is conceptualized as a care approach that recognizes patients as whole individuals. This perspective emphasizes the importance of acknowledging their lived experiences, dignity, agency, relationships, and embodied existence, rather than concentrating exclusively on disease or technical procedures. Dehumanization in health care is often an unintentional process in which patients are treated primarily as diagnoses, objects, or data, with reduced attention to their mental states and subjective experiences [8]. In this sense, it can function as an emotion regulation strategy for health care professionals under sustained stress, including in situations involving stigma, while also risking diminished patient autonomy and reduced care quality [8,16]. These concepts offer a valuable framework for analyzing how current changes in health care may enhance or diminish the human aspect of care.

To elucidate this analytical perspective, it is crucial to define patient-centered care, which implements many of these humanizing principles in clinical practice. This study conceptualizes patient-centered care according to the approach proposed by Rathert et al [3], which highlights care that respects patients’ values and preferences, responds to their needs, and fosters active patient involvement through effective communication, emotional support, and shared decision-making. These principles also align with the logic of 4P medicine, which emphasizes personalized, predictive, preventive, and participatory approaches to care. Rather than relying solely on syndrome-based treatment models, the 4P paradigm foregrounds early diagnosis, targeted intervention, illness prevention, and patient engagement, thereby reinforcing a holistic and proactive understanding of health and care [17].

Furthermore, as health care is increasingly shaped by digital technologies, these inquiries take on a distinct ethical dimension, as transparent policies, digital literacy, and ethical data governance are essential for public trust in AI-driven health care [18,19]. According to Floridi [20], digital ethics is defined as a practice-oriented framework that addresses the integration of ethical ideals, such as human dignity and accountability, in the design and implementation of digital technologies, rather than merely emphasizing their technical performance. This perspective corresponds with value-sensitive design in human-computer interaction, highlighting that AI systems are not value-neutral but incorporate ethical ideals such as dignity, autonomy, and accountability in their design, implementation, and usage [21].

Within this conceptual framework, the increasing integration of AI represents a significant development. While AI offers important opportunities to improve efficiency and precision in health care, it also raises questions about its potential to either rehumanize or dehumanize care. The growing emphasis on humanizing health care reflects the recognition that effective care involves not only clinical treatment but also empathy, trust, and meaningful relationships. Patients value personalized, compassionate care, while health care professionals seek supportive environments that enable well-being, professional development, and high-quality service [22]. Regarding rehumanization, a literature review by Busch et al [23] identified that an empathetic and respectful attitude toward patients, adequate human and material resources, and a balanced workload for health care providers are essential conditions for fostering meaningful, reciprocal patient relationships and delivering humanized care. In this context, the integration of AI has the potential to contribute to rehumanization by relieving clinicians of routine administrative tasks and enhancing diagnostic accuracy. These advancements can free up time for health care professionals to engage more deeply with patients, fostering communication, empathy, and trust. This renewed focus on the human dimension of care has the potential to improve both patient experiences and clinical outcomes [12]. Building on this perspective, AI-powered tools have been shown to support personalized communication by tailoring information and support to individual patient needs and preferences [11]. Additionally, AI holds promise for promoting health equity by improving access to care for underserved populations, thereby addressing systemic disparities in health care delivery [24]. Moreover, natural language processing and large language models can help translate complex medical terminology into more understandable information for patients, improving communication and supporting patient engagement and outcomes [25].

However, dehumanization has become a central concern in critiques of modern medicine, often linked to declining personal attention, emotional support, and relational care. Increasing reliance on technology, standardization, and efficiency may overshadow patients’ individuality and autonomy, reducing their role in care processes [26]. This shift has implications for both patients and health care providers. Health care providers may adopt dehumanizing practices as a means of emotional self-protection in the face of constant exposure to suffering and stress. While such strategies can enhance clinical performance by enabling psychological distance, they may also compromise empathy, communication, and trust, ultimately threatening the therapeutic alliance and the overall quality of care [8]. While AI can improve efficiency and diagnostic precision, its expanding role may reduce face-to-face interaction and risk treating patients as data points. Such dehumanization may negatively affect patient satisfaction, well-being, and the therapeutic alliance, highlighting the need to ensure that AI is designed and implemented in ways that support rather than replace the human elements of medical practice [27]. A nationally representative US survey found that although the public was generally optimistic about AI in diagnosis and treatment, concerns remained regarding misdiagnosis, privacy, costs, and reduced clinician-patient interaction. These concerns were particularly pronounced among racial and ethnic minority groups, highlighting the perceived risk of dehumanization in AI-driven health care [13]. Rony et al [14] highlight that concerns around dehumanization in health care often stem less from the use of AI itself and more from the potential erosion of personal connection in care. Their findings underscore the importance of integrating AI in ways that support, rather than undermine, empathetic and patient-centered care. Health care professionals, particularly nurses, are seen as essential in preserving compassion within increasingly digital environments, positioning AI as a tool that should enhance—not replace—human interaction [14].

As AI becomes increasingly integrated into health care, questions arise about its impact on the human dimension of care. While it offers substantial benefits, concerns remain that it may weaken empathetic, patient-centered interactions. Understanding whether AI contributes to the rehumanization or dehumanization of care requires examining not only the technology itself but also the broader context of its implementation across macrolevel (policy and infrastructure), mesolevel (institutional practices), and microlevel (individual behaviors and interactions). This multilevel perspective helps identify key enablers and barriers to humanized care in digital health care environments. According to Rosengren et al [15], from a person-centered care perspective, macrolevel policies and financing structures influence the prioritization of patient values and equity, mesolevel organizational cultures and care models dictate the implementation of these principles in practice, and microlevel clinician-patient interactions ultimately shape patients’ lived experiences of care. These multilevel dynamics become particularly visible in digital health contexts, where technological change intersects with existing system structures. In this regard, Asthana et al [28] assert that the implementation of eHealth innovations within the National Health Service in England is influenced by the interaction of macrolevel national digital policies, mesolevel organizational capacity and varying digital maturity, and microlevel acceptance by clinicians and patients. These multilevel challenges lead to ongoing inequalities and tensions in person-centered care and routine clinical interactions.

Expanding this viewpoint beyond digitalization, prior studies indicate that macro-meso-micro frameworks are essential for comprehending complex health care phenomena in a broader context. For example, Sawatzky et al [29] examined how response shift—changes in patients’ internal standards or values over time—affects the interpretation of patient-reported outcome measures. Their study highlighted how such shifts can influence clinical decisions at the microlevel, quality monitoring and accreditation at the mesolevel, and health policy and funding decisions at the macrolevel. Their multilevel framework illustrates how patient perceptions must be contextualized across different layers of health care to ensure valid, person-centered decision-making [29]. Similarly, Beirão et al [30] examined value creation in Portugal’s national electronic health record ecosystem, focusing on interactions among actors at the micro-, meso-, and macrolevels. Their findings show that although resource access, communication, and governance differ across levels, they remain interdependent, highlighting the need for coordinated system-wide efforts to improve patient experience and ecosystem viability [30,31]. Building on macro-meso-micro frameworks in digital health, this study extends prior research by examining how AI implementation influences rehumanization and dehumanization across system levels.

## Methods

### Research Design and Analytical Approach

The objective of this research is to examine the impact of AI implementation on the potential rehumanization or dehumanization of patient interactions, with a focus on the contextual factors that may influence these outcomes. Specifically, the study seeks to identify and analyze macrolevel (eg, health policy and technological infrastructure), mesolevel (eg, organizational strategy and culture), and microlevel (eg, individual attitudes and clinician-patient communication) determinants that mediate the relationship between AI integration and humanized care delivery. The research adopts a multilevel perspective with the aim of providing a nuanced understanding of how AI can be aligned with the principles of person-centered health care. In accordance with this purpose, the study examines the subsequent research questions:

- RQ1: How does the implementation of AI in health care shape the rehumanization or dehumanization of patient-provider interactions, and through which contextual factors do these effects emerge?
- RQ2: What macrolevel (eg, policy, regulatory, and infrastructural), mesolevel (eg, organizational strategies and cultures), and microlevel (eg, professional attitudes and clinician-patient communication) factors influence these processes?

To address the research questions, a qualitative study was conducted using semistructured interviews with a diverse group of experts from the health care sector. The objective of this study was to obtain an in-depth and comprehensive understanding of perspectives on the integration of AI in health care. Since this is a comparatively novel and developing field, a qualitative approach is especially pertinent, as it facilitates the exploration of emerging experiences, perceptions, and systemic dynamics that are not yet comprehensively understood or readily captured by quantitative methods [32].

Building on this rationale, this study used a grounded theory method due to its exploratory nature and the limited empirical research on AI’s role in the rehumanization or dehumanization of patient-provider interactions. This methodology provides a methodical approach to deriving theoretical ideas directly from the data, rendering it especially appropriate for environments with limited established frameworks. Grounded theory, as initially described by Glaser and Strauss [33], aims to construct a thorough, data-driven comprehension of a phenomenon via recurrent cycles of data collection and analysis. Strauss and Corbin [34] assert that the primary objective of this methodology is to generate conceptual elucidations derived from the empirical data, facilitating the concurrent evolution of the theoretical framework alongside the research process. This study used an inductive approach to identify key categories and establish theoretical connections when patterns emerged from the qualitative data [35].

Consistent with grounded theory principles, data collection and analysis occurred simultaneously, with insights from first interviews informing later inquiries. Theoretical saturation was considered achieved when no new categories or significant qualities arose from further interviews and when the evolving themes exhibited adequate consistency and explanatory power across cases. To improve the transparency and rigor of the analytical process, the study used the Gioia technique [36], which offers a systematic framework for grouping first-order concepts, second-order themes, and emergent aggregate dimensions. This integration of iterative inquiry and systematic coding guaranteed that the theoretical contributions were firmly grounded in participants’ narratives while maintaining rigorous qualitative research standards. The reporting of this qualitative study was guided by the COREQ (Consolidated Criteria for Reporting Qualitative Research) 32-item checklist to enhance transparency and completeness (Checklist 1).

### Data Collection

To identify appropriate participants for the research, purposive sampling was used, which is a widely used technique in qualitative research. The selection of individuals capable of providing profound, contextually grounded insights into the phenomenon under study is a fundamental aspect of this approach, and the recruitment process therefore followed a systematic procedure. Participants were identified through professional networks, health care institutions, and expert organizations across both the public and private sectors. Potential interviewees were contacted directly via email based on their documented expertise, professional roles, and involvement in AI-related health care activities. Recruitment and scheduling were coordinated individually, with interviews conducted either at participants’ workplaces or via secure online platforms, depending on their preference. The emphasis was on ensuring the relevance and depth of the data collected [31].

In this study, participants were selected based on their expertise in AI and health care, their availability and willingness to participate, and their ability to articulate their perspectives in a reflective and meaningful way. To address the research questions and examine the macro-, meso-, and microlevel factors influencing the processes of rehumanization and dehumanization in health care, this study used a qualitative research design based on 20 semistructured interviews. It was imperative that interviewees possessed both theoretical knowledge and practical experience with AI in health care. Of particular importance was the inclusion of stakeholders from both private and public health care institutions, ensuring that the research captured the complexity and diversity of the sector. While the participants were based in Hungary, their professional backgrounds included substantial international experience in European health care systems and, in a few cases, experience in US health care, which contributed to the richness of the perspectives that were gathered. This sampling process, consistent with grounded theory’s iterative and theoretically driven logic [36], ensured the inclusion of information-rich experts whose insights could meaningfully inform the emerging conceptual understanding of AI implementation. All interviews were conducted jointly by both authors, who also collaborated closely throughout the data analysis process, including coding, theme development, and interpretation.

Prior to conducting the interviews, an interview guide was developed. This guide delineated the primary research questions and thematic areas to be explored, as detailed in Multimedia Appendix 1, where the questions are categorized by macro-, meso-, and microlevels of study. The questions were customized for each participant’s professional background and expertise to ensure the discussion accurately represented their personal experience and domain knowledge. As posited by Holloway and Galvin [37], such a guide proffers a structured framework to support the researcher while allowing for flexibility; it is not intended to be followed rigidly, but rather to facilitate the collection of comparable information across participants while enabling in-depth exploration of the research questions. The interviews were conducted between July and August 2025. It is imperative to note that all interviews were audio-recorded with the informed consent of the participants, who were thoroughly briefed on the purpose of the research and the procedures for data handling.

### Ethical Considerations

This research was performed in compliance with recognized ethical standards for studies involving human participants. Ethical approval was secured by the Research Ethics Committee of Corvinus University of Budapest, Vice-Rector for Faculty and Research (approval number: KRH/173/2025, dated June 30, 2025). The committee evaluated the study design, data collection methodologies, and data management strategy of the research.

All participants were provided with comprehensive information regarding the study’s objectives, the voluntary nature of their involvement, the interview protocols, and the planned application of the findings. Informed written consent was acquired from all participants prior to their involvement, encompassing permission for audio recording. Participants were notified of their entitlement to withdraw from the study at any moment without justification and without facing any negative repercussions.

Participant privacy and confidentiality were meticulously safeguarded during the research procedure. Interview data were processed in an anonymous and nonidentifiable manner. Personal identifications were eliminated during transcribing, and participants were allocated distinct codes to ensure anonymity. Audio recordings and transcripts were securely saved and accessible exclusively by the study team. Results are presented in an aggregated format to guarantee the anonymity of individual participants. Participants did not receive any financial or nonfinancial compensation for their participation in the study.

### Data Analysis

Using the methodological framework of Gioia [36], a structured analysis was carried out to understand and generalize the empirical data. This methodology facilitated the recognition of first- and second-order concepts and the formulation of aggregate dimensions that encapsulate the interconnections among nascent themes [36,38]. The analysis was consistently informed by the core principles of grounded theory articulated by Glaser and Strauss [33], ensuring that the evolving theoretical findings were securely rooted in participants’ narratives.

The analysis commenced with open coding of the interview transcripts using NVivo software (Lumivero). The initial coding closely adhered to the terminology used by the experts, encapsulating their perspectives on AI’s capacity to rehumanize care, its potential for dehumanization, and the contextual elements influencing both outcomes.

In second-order coding, these initial categories were subsequently analyzed, developed, and interpreted using pertinent theoretical frameworks, such as patient-centered care, sociotechnical systems theory, and organizational transformation within health care. This phase facilitated the transition from descriptive findings to the development of profound conceptual patterns elucidating how macro-, meso-, and microlevel factors influence the human impact of AI in clinical practice.

The second-order themes were synthesized into aggregate dimensions through iterative comparison and theoretical elaboration, highlighting the dynamic interplay among structural variables, institutional determinants, and human experiences. In total, the analysis resulted in the identification of 8 macrolevel, 5 mesolevel, and 7 microlevel factors that collectively shape the potential for AI to foster either rehumanizing or dehumanizing trajectories in health care. These dimensions establish the foundation of a preliminary conceptual model that demonstrates how AI adoption might result in either rehumanizing or dehumanizing results, along with the mechanisms by which these processes occur within health care systems.

## Results

### Participant Characteristics and Overview of Findings

A total of 20 semistructured interviews were conducted with stakeholders representing multiple segments of the health care ecosystem. The sample included physicians and clinical specialists, several of whom also held leadership or managerial roles, alongside participants with backgrounds in health care management and policy, health economics and academic research, legal and regulatory fields, and industry and AI development. These overlapping professional roles provided a multistakeholder perspective on the implementation of AI in health care. Participants represented both public and private health care contexts and varied in their level of AI involvement, ranging from extensive implementation or development experience to indirect exposure or no direct use. The sample included participants identifying as women and men. Gender was recorded as a self-reported descriptive characteristic and was not examined as an analytical variable. Interview duration ranged from 45 to 67 minutes. Across stakeholder groups, the core themes regarding the rehumanizing and dehumanizing potential of AI were largely consistent, although participants emphasized different aspects depending on their professional background. Detailed participant characteristics are provided in Multimedia Appendix 2.

The results section presents the interview findings on how AI integration may contribute to rehumanization or dehumanization in health care. In total, the analysis identified 8 macrolevel, 5 mesolevel, and 7 microlevel factors that collectively shape these trajectories, highlighting how policy environments, organizational practices, and interpersonal dynamics interact in determining AI’s human impact.

### Rehumanization Aspects of AI in Health Care

Across interviews, participants emphasized that the quality of patient interaction, effective patient management, and clear communication are central to clinical outcomes and patient satisfaction. Several interviewees described the doctor-patient relationship as a cornerstone of patient-centered care, while noting that growing administrative burdens and fragmented information systems increasingly limit the time available for meaningful interpersonal engagement.

*Currently, in healthcare, I see that doctors spend a lot of time trying to interpret medical documents or analyze accumulated data. Whenever a patient arrives, they spend less time with them and more time reviewing their medical history*.

[Participant 1]

In response to these challenges, participants frequently framed the rehumanization of health care as an important goal, emphasizing the need to restore the relational and ethical core of medical practice. AI was often described as a “double-edged sword”: while poorly implemented technologies may intensify depersonalization, thoughtfully designed systems may strengthen the human dimensions of care. Thematic analysis identified three main categories of AI-enabled interventions associated with rehumanization: (1) tools supporting health care professionals, (2) tools enhancing patient autonomy and engagement, and (3) hybrid solutions addressing both.

Participants most frequently associated the rehumanizing potential of AI with its ability to reduce clinicians’ administrative burden. Systems that transcribe consultations, generate structured documentation, and retrieve relevant medical histories may allow physicians to devote more time to direct patient interaction. Instead of navigating electronic health records or writing discharge notes, clinicians can focus more on listening to patients, explaining diagnoses, and cocreating care plans. AI-powered summarization of clinical literature and patient data can also support more informed decision-making in real time.

Participants also highlighted patient-facing AI tools as an important means of improving communication and engagement, particularly by transforming how health information is delivered and understood. Many medical documents are written in complex clinical language that is difficult for nonspecialists to interpret. Large language models can simplify diagnostic reports, provide plain-language explanations of laboratory results, and respond to patient questions conversationally. These functions can improve health literacy, reduce knowledge asymmetries, and foster greater inclusion in the care process, particularly for patients managing chronic or complex conditions.

AI was also described as offering scalable solutions for patient education. Conversational agents providing information about preventive screening or disease management (eg, cervical cancer or diabetes) can reach large populations without increasing clinicians’ workload. This may be especially valuable in resource-constrained settings or underserved communities where access to direct clinical guidance is limited.

*Solutions are now available that simplify results while providing very empathetic and sympathetic responses, which I believe makes care very patient-centered. And this is precisely the area where healthcare workers generally lack capacity due to their heavy workload*.

[Participant 2]

Participants also noted that patients with complex diagnoses or intensive treatment regimens are often overwhelmed by the volume of information they receive. AI tools can structure treatment plans, medication schedules, and follow-up instructions, allowing patients to review them at their own pace. This can improve understanding, support emotional processing, and strengthen trust, particularly during vulnerable periods. While such tools cannot replace human emotional support, they can enhance the continuity and clarity of communication.

Hybrid AI applications supporting both patients and clinicians were also frequently mentioned. Digital preconsultation tools can structure medical histories and family background information, providing clinicians with concise summaries that enable more focused consultations while helping patients reflect on relevant symptoms and contextual factors. However, without careful workflow integration and adequate user education, such systems may increase communication burdens, particularly in resource-constrained primary care settings.

AI can also support remote patient management through telemonitoring and virtual follow-up platforms, enabling symptom tracking, recovery monitoring, and automated guidance while alerting clinicians to potential concerns. These systems can enhance continuity of care, patient trust, and transparency by supporting appointment scheduling, reminders, and care navigation. Nevertheless, excessive automation risks undermining interactions perceived by patients as inherently human, especially at critical decision points.

*I believe that the personal relationship between doctor and patient should not change. I think it is very important from both the patient’s and the doctor’s point of view, but I believe that telemedicine would play a very important role in such pre-screening, where there would be a virtual connection and AI could make a preliminary diagnosis*.

[Participant 1]

### Dehumanization Aspects of AI in Health Care

Participants also highlighted potential dehumanizing effects associated with the integration of AI into health care. While AI can improve efficiency, optimize workflows, and enhance diagnostic precision, its growing role also raises social and psychological concerns. Chief among these is the risk of dehumanization, whereby patients and clinicians may experience a loss of individuality, agency, or relational connection as reliance on automated systems increases.

From an organizational and systemic perspective, several participants identified the economic logic underlying AI deployment as a key driver of potential dehumanization. In many health care systems, AI is primarily introduced to reduce costs, optimize resources, and improve efficiency. While these objectives are understandable from a management perspective, they may prioritize throughput over therapeutic relationships. In such contexts, both patients and clinicians risk being reduced to variables within process optimization.

From the patient perspective, interviewees emphasized the erosion of personalized attention as a central concern. Although AI may enhance diagnostic precision and procedural efficiency, increasing mechanization—particularly in automated screening or intervention processes—may reduce opportunities for meaningful interaction with health care professionals. For many patients, the therapeutic relationship itself contributes to healing; the absence of consultation, explanation, or emotional support may lead to dissatisfaction even when clinical outcomes are positive. Physicians are therefore expected to fulfill not only biomedical roles but also psychosocial ones, offering reassurance, guidance, and human presence.

*I believe that the doctor’s personal diagnosis and treatment plan are very important, because they often have the same effect on patients as a psychologist*.

[Participant 15]

Limited consultation time may further contribute to frustration, particularly in private health care settings where patients often associate payment with receiving attention and personal interaction. Even when AI-supported treatments produce positive results, a lack of interpersonal engagement may be interpreted as indifference or negligence, highlighting the gap between clinical efficiency and patient experience.

*In private healthcare in particular, the time spent with the doctor is important to patients. If the doctor spends little time with the patient, they will be dissatisfied and even negative, even if the results are good. They need consultation and conversation, otherwise they will be disappointed*.

[Participant 16]

Participants also raised concerns about the impact of AI on health care professionals, particularly regarding professional identity and authority. As AI systems demonstrate competence in tasks traditionally associated with expert clinical judgment, physicians may experience status anxiety or a perceived erosion of professional value. If diagnostic or decision-making responsibilities increasingly shift to automated systems, this may affect morale, job satisfaction, and the long-term attractiveness of the profession.

Another concern relates to overreliance on automated decision-making. If AI systems begin to guide clinical decisions without sufficient human mediation or transparency, patients may experience confusion, mistrust, or resistance, particularly when they cannot understand how recommendations are generated. Current AI models are primarily designed to support clinicians rather than replace them; however, poorly framed or opaque AI-driven recommendations may still be perceived as intrusive or coercive.

At present, AI is largely introduced to support overstretched health care systems and mitigate workforce shortages rather than replace core clinical roles. Nevertheless, as technological capabilities advance, the balance between human and machine involvement may shift, raising questions about the future role of health care professionals and the preservation of human dignity in care.

*Personalization is important to patients. AI can improve quality in some areas, such as diagnosis, but excessive mechanization (surgery, screening) leads to less contact with doctors, which may result in patient dissatisfaction*.

[Participant 16]

Overall, participants emphasized that while AI is frequently presented as a solution to systemic inefficiencies, its implementation must be carefully managed to avoid unintended dehumanizing consequences. Technological advancements should complement rather than replace the relational and interpretive dimensions of medical practice. Maintaining this balance was considered essential to ensuring that health care remains not only efficient but also humane.

### Multilevel Factors Shaping Rehumanization and Dehumanization in AI-Enabled Health Care

#### Overview of Interacting Macro-, Meso-, and Microlevel Determinants

**Figure 1.**
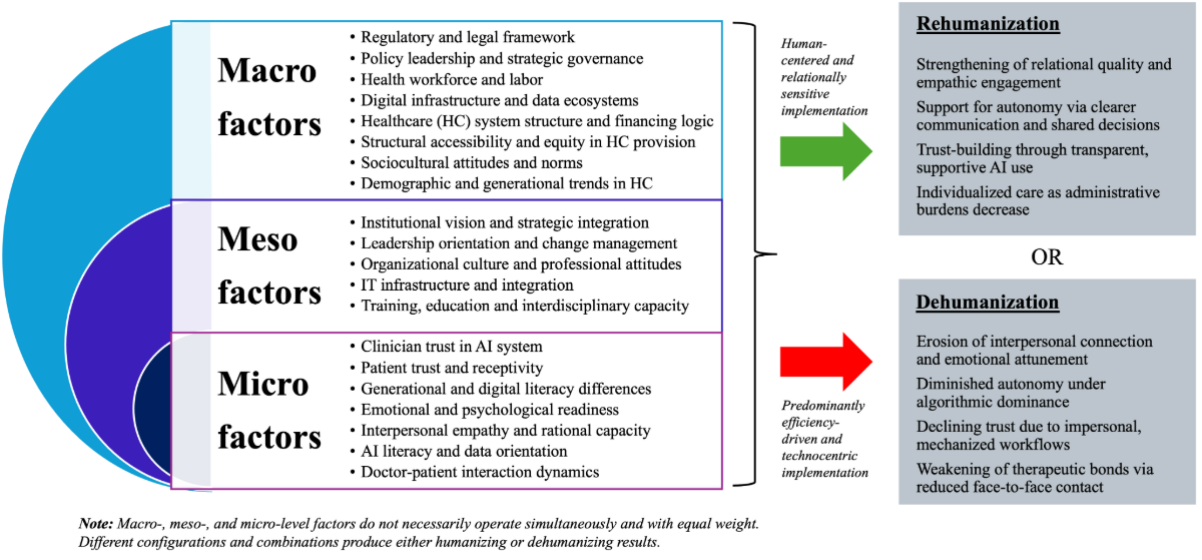
Macro-, meso-, and microlevel factors shaping rehumanizing and dehumanizing trajectories in AI-integrated health care. AI: artificial intelligence.

Drawing on the interviews, this section reviews the macro-, meso-, and microlevel factors that may influence the rehumanizing or dehumanizing effects of integrating AI into health care. These factors span structural, organizational, and individual domains, and their interactions are often complex and interdependent. It is important to note that changes at one level can generate cascading effects across other levels, which can amplify or reduce their overall impact on the nature and quality of patient care. Figure 1 provides an overview of the identified macro-, meso-, and microlevel factors and illustrates how these elements, in combination, may shape trajectories toward either rehumanizing or dehumanizing outcomes in health care.

#### Macrolevel Factors

The broader systemic environment in which AI is implemented plays a decisive role in shaping its potential to foster rehumanization or to perpetuate dehumanizing effects. A range of macrolevel factors (see Multimedia Appendix 3)—spanning regulatory and legal structures, policy leadership, workforce capacity, digital and data infrastructures, health care financing models, accessibility, sociocultural norms, and demographic trends—collectively influence the conditions under which AI adoption occurs and the equity, sustainability, and human-centeredness of its outcomes.

The *regulatory and legal framework* encompasses laws, ethics, and institutions related to health care AI. Alongside sector-specific legislation, it encompasses data protection frameworks such as the General Data Protection Regulation and AI governance mechanisms, including the EU AI Act. This framework establishes the legal and ethical boundaries within which AI systems can be integrated into health care, thereby shaping the scope and nature of their application.

*The EU AI Act, which is otherwise a very good, comprehensive and forward-looking piece of legislation*.

[Participant 12]

*And now the European Health Data Space is also being developed, with the aim of enabling the transfer of health data across national borders*.

[Participant 7]

Effective regulation can safeguard patient data, ensure ethical oversight, and protect vulnerable populations, thereby strengthening trust in AI adoption. Clear legal frameworks promote accountability and transparency while helping align AI use with patient-centered values rather than purely operational or commercial priorities. However, overly rigid regulations may limit context-sensitive AI implementation, particularly in rapidly evolving health care environments. Legal uncertainty—such as unclear liability rules or inconsistent national policies—can delay adoption or encourage excessively cautious use. In addition, compliance requirements may divert institutional resources toward administrative tasks, potentially increasing workload pressures on health care providers.

*Policy leadership and strategic governance* delineate the prioritization, coordination, and financing of health care AI deployment by political institutions. This includes national strategies, dedicated government bodies, and long-term investment policies to develop and integrate AI. Political considerations influence the use of AI in health care systems to either enhance equity, quality, and innovation or to constrain costs and optimize operational efficiency.

Strategic coordination across ministries, agencies, and sectors reduces redundancy and promotes scalable, coherent health care ecosystem execution. Clear communication of policy objectives facilitates stakeholder engagement and equips society for the transformative role of AI in health care. A government focused on cost containment may portray AI as reducing the workforce, reducing the human element of care delivery. Politically exploiting AI for immediate benefit can hurt patient-centered innovation and confidence. Disjointed implementations may fail to scale and exclude key stakeholders from decision-making without a strategic vision.

*Health care workforce and labor market dynamics* refer to the availability, distribution, skills, and well-being of health care personnel, as well as broader labor market trends within the sector. These include workforce shortages, the undercompensation of support staff, and generational differences in professional values and expectations. Such conditions shape whether AI is perceived as a tool that supports health care professionals or as a potential substitute for them.

By reducing administrative tasks and cognitive workload, AI can allow clinicians to devote more time to relational aspects of care, potentially improving patient interaction and professional satisfaction. In resource-constrained settings, AI may also support diagnostic accuracy and therapeutic decision-making, enabling more timely and effective care. However, if AI is used to replace rather than assist clinicians, it may weaken human interaction and the therapeutic relationship. The perceived devaluation of professional expertise due to automated decision-making may also contribute to disengagement, reduced morale, and resistance to innovation. In extreme cases, excessive reliance on algorithmic decision support may limit professional judgment and contextual adaptation, thereby constraining personalized care.

*We see that it is simply necessary to accept in the clinical field that their future will be determined by their attitudes towards technology and their cooperation with technology, and new professions will be created.*

[Participant 9]

At the national level, *digital infrastructure and data ecosystems* provide the technical and organizational foundations for digital health and AI-specific functionalities, including interoperable electronic health records, secure data exchange frameworks, and advanced computational resources such as national cloud services and large-scale health data repositories. Differences between well-resourced, integrated national systems and underfunded, fragmented ones directly shape the feasibility and quality of AI implementation across the health care sector.

AI can be effectively incorporated into workflows within centralized, interoperable national health information systems, thereby reducing care fragmentation and enabling physicians to prioritize patient engagement over administrative tasks. Efficient access to comprehensive, longitudinal patient records enhances trust and continuity in multidisciplinary and cross-regional contexts. Harmonized national data ecosystems facilitate early detection, enable proactive public health interventions, and provide tailored decision support. Fragmented national IT infrastructures compel health care providers to manage various systems, resulting in decreased efficiency and diminished patient-centered communication.

*Three factors are necessary for AI-based developments: sufficient quantity and quality of available data, interconnected central systems, and data science capacity, including trained physicists, engineers, data scientists, and clinicians*.

[Participant 9]

The *structure and financing logic of health care systems* shape how care is organized and funded at the macrolevel, including whether systems are centralized or market-driven, publicly funded or privately operated. These arrangements—through national insurance schemes, private insurers, or hybrid models—influence how AI is adopted, who benefits from its use, and whether priorities focus on equity, quality, efficiency, or profitability.

In centralized, publicly funded systems, AI can support equitable access, continuity of care, and population health management. Insurance models that emphasize prevention and long-term outcomes may facilitate AI-supported early detection, chronic disease management, and personalized care planning. Data-driven resource allocation can further improve efficiency while promoting fair distribution of services. In market-oriented or predominantly private systems, AI adoption is more often driven by cost containment and service standardization. Access to advanced AI-supported care may become concentrated among higher-income or digitally literate populations, potentially widening disparities. Profit-oriented reimbursement structures may also deprioritize complex or less profitable cases, reinforcing existing inequalities.

*Structural accessibility and equity in health care provision* influence the accessibility of health care for individuals and communities. The geographic distribution of health care facilities, the balance between public and private sectors, insurance coverage, affordability, and infrastructure availability extend beyond just digital access. The beneficiaries of AI-enhanced health care are contingent upon these structural circumstances. In regions with restricted in-person health care, AI can facilitate remote access for telemedicine, virtual consultations, and remote diagnostics. The introduction of public AI can mitigate systemic disparities in service distribution by using new technology in areas of need rather than in locations of maximum profitability. Moreover, algorithmic triage and resource allocation might enhance fairness in high-demand situations by prioritizing instances with the greatest necessity, thus enhancing efficiency without sacrificing equality. If AI deployment adheres to a market-driven model, benefits may be concentrated in affluent metropolitan regions, exacerbating the accessibility gap.

*Sociocultural attitudes and health norms* reflect prevailing cultural values that shape how health care is perceived and delivered, including views on medical authority, patient autonomy, and shared decision-making, as well as whether health is understood as a personal responsibility or a collective right. When developed inclusively, AI can enhance cultural awareness, tailor care to diverse societal values, and help reduce stigma associated with mental health and chronic illness. Sociotechnical integration—aligning technological design with social and cultural contexts—may enhance patient autonomy and mitigate power imbalances in physician-centered systems, enabling more participatory and collaborative care. However, standardized AI protocols may conflict with diverse cultural perspectives on health, aging, and mortality, while algorithmic biases and excessive uniformity risk reinforcing cultural exclusion, overlooking relational and symbolic aspects of care, and contributing to the commodification of health care in highly individualistic systems.

*Demographic and generational trends* shape health care demand, service expectations, and the adoption of medical technologies. Population aging increases the need for long-term care, chronic disease management, and personalized support, while generational differences in digital literacy and attitudes toward autonomy and professional authority influence how AI is used across patient groups. AI technologies may help address complex care needs associated with aging populations, including multimorbidity and cognitive decline, and support aging in place through remote monitoring and home-based care. Designing AI systems with generational inclusivity in mind can reduce barriers to access and help sustain autonomy in later life, while digitally fluent younger cohorts may facilitate more participatory care models. However, AI systems that do not account for cognitive, sensory, or functional limitations may be inaccessible for older adults. Replacing human care with AI in eldercare settings may also risk social isolation and emotional neglect. Age-related bias in data or system design may further marginalize older adults, while generational mistrust of technology may limit engagement with AI-supported care.

*Very often, technical limitations are the problem, with the older generation lacking basic computer skills. Middle-aged and older generations working in healthcare are unaware of the possibilities available to them*.

[Participant 6]

#### Mesolevel Factors

The institutional context in which AI is introduced to the health care sector is crucial in determining whether such technologies promote rehumanization or reinforce dehumanizing tendencies. A variety of mesolevel factors (see Multimedia Appendix 4), including institutional strategy, leadership, organizational culture, IT infrastructure, and training ecosystems, influence how AI is adopted, integrated, and experienced within clinical settings.

One such factor is the *institutional vision and strategic integration* of AI. This pertains to the extent to which health care organizations use AI in their strategic, operational, and innovative objectives. This examines whether AI is used to resolve clinical and systemic challenges or merely as a trend. AI solutions that enhance clinician-patient engagement, diminish administrative burdens, and augment access to care are more likely to be adopted in patient-centered institutions.

Public hospitals, academic medical centers, and private clinics exhibit significant variations in their institutional methodologies and health care provision. Academic institutions possess numerous financing avenues and robust research environments, whereas public hospitals sometimes contend with constrained finances and restrictions. This enables them to adopt AI in an ethical and empirical manner. Conversely, private clinics may emphasize efficiency, competitiveness, and patient satisfaction, perhaps fostering innovation and a focus on short-term implementation strategies.

AI may be implemented in institutions reactively, motivated by perceived innovation pressures rather than genuine therapeutic requirements, and without a strategic framework. Ad hoc or shallow adoption typically results in fragmented implementation, misaligned use cases, and system redundancy. AI technologies may lack meaningful integration into care operations without defined objectives, human engagement, or responsibility. Misaligned institutional metrics and incentives constitute another challenge. When performance metrics emphasize throughput, cost reduction, or digital adoption over care quality, AI is used to achieve operational objectives rather than address human requirements. Even in circumstances where human presence is essential for establishing trust, communication, and developing a therapeutic alliance, automated systems may supplant interpersonal care.

*In my opinion, AI will essentially be a tool that changes at an incredible pace, with new solutions and opportunities emerging every week. I believe that incorporating related tasks into the institution’s strategy is important. It is not certain that anyone needs to develop a separate AI strategy, but it is important to incorporate it*.

[Participant 13]

Another key mesolevel factor shaping the human impact of AI implementation is *leadership and change management*. This refers to how institutional leaders assess, prioritize, and guide the implementation of AI, including their openness to innovation, responsiveness to staff feedback, and ability to manage organizational change. Leadership also influences resource allocation and determines whether AI is framed as a collaborative clinical support tool or primarily as an efficiency-driven directive.

Visionary and inclusive leadership can position AI as a means of strengthening human-centered care. Leaders who support iterative learning, pilot initiatives, and ethical reflection create opportunities for staff to contribute to implementation processes and raise concerns. Training, stakeholder engagement, and phased implementation can foster shared ownership and trust, enabling context-sensitive AI integration that supports clinical values and continuity of care. Conversely, top-down implementation that excludes clinical staff may undermine legitimacy and provoke resistance or superficial compliance. When leaders focus primarily on efficiency or innovation metrics, the ethical, relational, and psychological implications of AI adoption may be overlooked, weakening long-term acceptance and institutional trust. Rapid deployment without adequate change management may also disrupt workflows, blur responsibilities, and erode confidence in both the technology and leadership.

The *organizational culture and professional attitudes* significantly impact the adoption and experience of health care AI. This encompasses collective beliefs and norms on innovation, hierarchy, care provision, and the impact of AI on professional identity. In cultures that prioritize open discourse, critical analysis, and employee-driven innovation, AI can augment clinical judgment rather than undermine it. Professionals are more likely to engage when their expertise is acknowledged, and adoption processes align with their lived experiences. Recognizing the diverse experiences of individuals across various occupations and levels of seniority mitigates the risk of professional marginalization. Conversely, distrust, superficial implementation, and rigid hierarchies may engender skepticism or defensiveness toward AI. AI may be opposed or used superficially if perceived as a threat to clinical autonomy or a cause of deskilling. AI may devolve into a superficial technology, diminishing care quality and employee morale.

*Our colleagues are open to technology, competitiveness is important to them, so they have to adopt new solutions, be open-minded and follow trends. They consider digitalization important, and younger employees are generally more open to digital and AI-based solutions*.

[Participant 16]

The implementation of AI in health care also depends on institutional *IT infrastructure*, including system interoperability, legacy software, device compatibility, data capacity, and integration with national or regional platforms. Differences between well-funded, integrated systems and fragmented, under-resourced environments significantly affect the feasibility and quality of AI adoption.

*If a unified healthcare IT system were to be introduced nationwide, data would be much easier to access. We often encounter patients who have already been treated by three, four, five or even six different healthcare providers, and gathering all of their data is a particularly lengthy process*.

[Participant 6]

In interoperable digital environments, AI can be integrated into clinical workflows to enable faster information retrieval, reduce administrative burdens, and support patient-centered care. Cloud-based solutions may further expand access to advanced AI tools without overloading local infrastructure, particularly in resource-constrained settings, provided that appropriate regulatory safeguards and data protection mechanisms are in place to ensure safe and equitable implementation. Conversely, outdated systems and poor interoperability may fragment data, disrupt care continuity, and increase technological complexity for clinicians. Such conditions can limit the effective use of AI and shift attention away from patient interaction. Overall, IT infrastructure plays a decisive role in determining whether AI supports more human-centered care or contributes to digital detachment.

The knowledge, application, and use of AI are also contingent upon the ability of health care facilities and universities to provide relevant *education, training, and interdisciplinary capacity*. This includes technical upskilling for clinical staff as well as academic programs that promote ethical awareness, digital literacy, and collaboration across medical, technological, and policy domains. Institutions that invest in integrated and interdisciplinary education are better positioned to support socially responsible AI implementation. Incorporating critical AI literacy into university curricula helps future professionals engage with AI reflexively and prioritize human values in technology design and use. Continuous and inclusive training for health care professionals can further ensure that AI supports rather than replaces human judgment and empathy. Conversely, technocentric or narrowly focused training may lead to overreliance on AI or resistance due to limited understanding. Educational inequalities between institutions or regions may also reinforce systemic disparities by concentrating AI literacy and influence in already well-resourced settings.

#### Microlevel Factors

At the microlevel, the interactions, perceptions, and capabilities of individual stakeholders are pivotal in shaping the experience of AI in health care. Factors such as clinician and patient trust, generational and digital literacy differences, emotional readiness, interpersonal empathy, AI literacy, and the dynamics of doctor-patient relationships (see Multimedia Appendix 5) directly influence whether AI integration enhances or undermines the human connection in care delivery.

*Clinician trust* in AI systems is a key microlevel factor shaping the human impact of AI in health care, referring to health care professionals’ confidence in the reliability, clinical relevance, and ethical integrity of AI tools. This trust is influenced by factors such as prior technological experience, alignment with clinical judgment, system transparency, and perceived implications for professional identity. When clinicians are involved in the development or adaptation of AI tools, they are generally more willing to adopt them. Transparent and explainable systems enable professionals to evaluate AI outputs and integrate them into clinical reasoning, thereby strengthening confidence. AI that supports rather than replaces medical decision-making can reinforce clinical authority and encourage responsible use.

Conversely, limited transparency may undermine trust, particularly when clinicians cannot understand how recommendations are generated. If AI is perceived as replacing rather than supporting expertise, it may provoke resistance, disengagement, or underuse. The introduction of AI without clinician involvement can also threaten professional identity and reduce acceptance. Ultimately, clinician trust develops through experience with AI implementation and plays a decisive role in determining whether AI strengthens clinical practice or undermines professional autonomy and confidence.

*I think the biggest obstacle to trust at the moment is that practical experience and success with AI solutions is still limited, with the exception of one or two areas, such as imaging diagnostics. I think this is exactly what centralized implementation could help with: a centralized pilot in one or two areas, so that everyone is involved to some extent and can see that this really can be a credible system*.

[Participant 1]

*Patient trust and receptivity* reflect patients’ readiness to engage with AI-supported health care. This trust is shaped by communication quality, transparency regarding the use of AI, prior digital experience, and expectations about human involvement in care. When health care professionals clearly and empathetically explain how AI is used, patient autonomy, informed consent, and engagement may increase. Familiarity with consumer AI technologies, such as chatbots or recommendation systems, may also normalize AI in health care. The accuracy and personalization of AI-supported services can further enhance their perceived legitimacy and encourage patient participation.

Conversely, limited transparency or impersonal use of AI tools may weaken patient trust, particularly if technology replaces human interaction or its role remains unclear. Patients may evaluate AI systems more critically than human clinicians, expecting consistently high levels of accuracy and objectivity. While human errors are often interpreted within a relational context, even minor AI errors may provoke skepticism. In addition, digital exclusion or limited digital literacy—often linked to socioeconomic status, age, or education—may lead to alienation and reduced confidence in AI-supported care.

*Differences in generational and digital literacy* encompass digital competence, receptiveness to innovation, and technological familiarity, frequently influenced by age and exposure to digital tools. These differences influence the adaptation of health care workers and patients to clinical AI systems. Younger, digitally proficient users might facilitate AI adoption by normalizing new technology and assisting those who are less technologically inclined. Systematic and focused training can mitigate digital exclusion and assist older or less experienced users in effectively using AI-assisted care. Restricted digital competencies among older professionals or patients may result in social isolation or a passive reliance on others for technological navigation, thus constraining autonomy and self-assurance. Access to digital resources, such as high-speed internet and suitable devices, may exacerbate socioeconomic and health disparities, leading to unequal participation with AI-enabled services.

The *emotional and psychological preparedness*—comprising emotional condition, cognitive capacity, and willingness to change—affects the use of AI technologies by health care professionals and patients. Burnout, stress, and work-life balance can influence motivation, flexibility, and the willingness to adopt new tactics. When used to alleviate administrative burdens, AI solutions can diminish emotional fatigue, enhance professional motivation, and optimize work-life balance. Framing AI as a supportive rather than a disruptive element in practice can facilitate its acceptance as an ally in care delivery rather than a menace to autonomy or competence. Health care professionals frequently experience burnout or emotional fatigue because of excessive administrative burdens or staffing constraints, which can impede learning and adaptability, resulting in avoidance or resistance. AI systems that introduce complexity, diminish control, or undermine professional autonomy may heighten stress and disengagement. Individuals may emotionally detach from innovation processes, perceiving AI as a hindrance rather than an asset.

*Interpersonal empathy and relational capacity* are key qualities in a clinician, describing their ability to communicate with empathy, understand patients’ perspectives, and sustain meaningful interpersonal relationships. Establishing trust, fostering patient engagement, and ensuring technology enhances rather than supplants human care necessitate these qualities.

*According to numerous studies, machines already produce much better results than humans in certain areas, but people still like to hear these results from human beings and believe them. That way, they believe them more readily*.

[Participant 13]

Clinicians may prioritize relationship-centered care by using AI-driven efficiencies such as automated documentation and data retrieval. Training in communication and empathy can assist physicians in preserving emotional attunement in digitally mediated environments. Clinicians managing decision-making guarantee ethical responsibility and relational confidence. However, assigning linguistic or emotional tasks to AI may diminish patient emotions. An excessive dependence on data-driven outputs may divert attention from patients’ narratives, values, and subjective experiences, which are essential for mutual comprehension and personalized care. Minimizing in-person interactions may undermine the therapeutic connection, weakening relational dynamics that enhance patient confidence.

Furthermore, *AI literacy and data orientation* refer to health care professionals’ ability to understand, critically evaluate, and appropriately use AI tools and data-driven outputs in clinical practice. This competence is essential to ensure that AI supports decision-making rather than being over-relied upon or dismissed. It includes understanding how AI systems function, recognizing their limitations, and interpreting their outputs in clinically and ethically appropriate ways.

Health care professionals with foundational AI literacy can critically assess AI recommendations, integrate them into evidence-based practice, and personalize care accordingly. A data-oriented approach can further support the effective use of patient information for informed decision-making. Institutional support through training and continuous education can strengthen professionals’ confidence and autonomy in using AI responsibly. Conversely, limited technical understanding may lead to inappropriate use, such as applying AI outputs without sufficient context or uncritically relying on automated recommendations. Uncertainty about how AI systems operate may also reduce professional confidence and hinder effective decision-making.

Finally, the *dynamics of doctor-patient interactions* encompass the communication, ethics, and power relations that influence how clinicians and patients exchange information, make decisions, and build trust. AI technologies have the potential to reshape these dynamics and transform clinical decision-making, information accessibility, and interpersonal standards.

*It is a serious challenge today that patients arrive at the doctor’s office already thinking they know more than their doctor, having looked things up on Google, ChatGPT, etc*.

[Participant 9]

When used thoughtfully, AI may simplify intricate medical information and promote collaborative decision-making. Using AI tools transparently during consultations demonstrates that technology enhances clinical judgment, hence fostering confidence. AI has the potential to distribute epistemic authority, diminishing hierarchical barriers and enabling patients to engage more actively in discussions regarding their care. If AI systems dictate all decisions, algorithmic authority may consolidate control, obscure accountability, and restrict patient involvement. Exclusion of patients from AI-driven decisions may undermine trust and autonomy. Furthermore, substituting dialogical engagement with automated methods may depersonalize the therapeutic alliance and diminish the essential human connection required for effective therapy.

## Discussion

### Principal Findings

This study represents one of the first empirical investigations of the potential rehumanizing and dehumanizing effects of AI integration in health care, examining how these outcomes are shaped by the interaction of macro-, meso-, and microlevel factors. By systematically mapping these influences, the research provides a multilevel perspective on the social, organizational, and ethical conditions that shape the practical use of AI in health care. These insights can inform policymakers, health care leaders, and clinicians on how to implement AI systems that are both operationally effective and aligned with patient-centered care. By highlighting both opportunities and risks, the study also contributes to anticipating and mitigating unintended consequences, helping ensure that AI strengthens rather than undermines the human dimensions of medicine.

Following a series of interviews, it was found that participants generally agreed that AI has significant potential to enable rehumanization in the field of health care. However, they emphasized that without a thorough understanding of the macro-, meso-, and microlevel factors identified in this study—and without deliberate alignment with patient-centered principles—the benefits of AI could easily be compromised or redirected toward efficiency goals that fail to enhance the patient experience. The findings reaffirm the widely acknowledged strengths of AI—its ability to improve diagnostic accuracy, optimize workflows, reduce administrative burdens, and enable more personalized, targeted treatments—while also making clear that these benefits will not be realized automatically. To achieve equitable and sustainable implementation, it is essential to engage in careful planning, structured education, robust support for patients and professionals, and adequate infrastructure. The interviews indicated variations among stakeholder groups. Senior physicians exhibited diminished trust in AI solutions, reflecting generational disparities at the microlevel, whereas legal experts and business consultants underscored the intricacies of regulatory frameworks, perceiving regulation as either a vital safeguard or a possible impediment to innovation; across various viewpoints, the incorporation of digital ethics and data protection was uniformly recognized as crucial.

Participants emphasized that AI could improve care quality, alleviate workforce shortages, and reduce burnout by shifting repetitive tasks away from clinicians. However, this raises fundamental strategic questions: how will the freed capacity be used? If regulations, institutional strategies, or governmental directives require the use of AI for rehumanization purposes, will this result in longer, more meaningful consultations and stronger follow-up, or will it simply normalize shorter patient interactions? The evolving role of medical assistants further illustrates the complexity of such changes. Assistants often contribute far more than technical support: they serve as witnesses during consultations, provide personal assistance, and support the relational aspects of care. Whether these roles are preserved, adapted, or eliminated depends on organizational priorities, budgets, and long-term strategies. The interviews also revealed the perception that technological advancement has diminished the authority of physicians in some contexts. This highlights the importance of preserving trust, professional identity, and interpersonal rapport in technology-rich care environments. Empathy was identified as another critical area for improvement: while certain specialties prioritize trust over explicit empathy, time saved by AI could be invested in rebuilding more meaningful patient-clinician relationships, particularly given the prevalence of burnout and reduced patient engagement. Currently, certain core aspects of care, such as physical examinations and direct patient contact, cannot be delegated to AI and still require skilled human involvement. However, future developments may alter this balance. Furthermore, time savings from automation will only translate into better care if they are deliberately structured to enhance the patient experience. In the absence of intentionality, efficiency gains may become detached from concrete enhancements in outcomes. Importantly, increased time availability alone does not automatically lead to stronger patient-centered care. While AI may create opportunities for more human interaction, patient-centered care requires a shared understanding of its principles, including meaningful communication and the cocreation of care, as well as organizational and professional conditions that enable clinicians to use this time in patient-centered ways.

In summary, this study’s contribution lies in providing one of the first empirically grounded frameworks for understanding how AI in health care can promote or hinder rehumanization across interconnected system levels. It shows that the potential of AI is not just a technical issue but also depends on strategic, ethical, and cultural factors. Realizing this promise will require coordinated action across regulatory frameworks, institutional strategies, governmental policies, workforce planning, and cultural adaptation, ensuring that technological progress serves as a catalyst for more human-centered, empathetic, and equitable health care.

### Theoretical Contributions

Building on established frameworks of humanization and patient-centered care [2,3], this study makes a substantive empirical contribution by demonstrating that rehumanization and dehumanization in AI-enabled health care are not inherent to the technology but are produced by concrete implementation choices and governance structures. While prior literature has highlighted the ethical and relational implications of AI in health care [8,26], the present findings clarify how these implications are enacted across macrolevel policy incentives, mesolevel organizational strategies, and microlevel clinical interactions. In doing so, the study advances patient-centered care and 4P medicine scholarship [17] by showing that personalization, participation, and relational quality are contingent achievements shaped by institutional priorities, leadership, and everyday clinical use, rather than automatic consequences of AI adoption.

### Practical and Policy Implications

The findings indicate that the influence of AI on rehumanization or dehumanization is significantly determined by policy and leadership decisions, rather than by technology in isolation. At the macrolevel, national AI and digital health strategies must incorporate human-centered values, ensuring that regulatory frameworks, funding mechanisms, and performance indicators facilitate patient-centered care, equity, transparency, and human oversight in conjunction with efficiency. At the mesolevel, hospital leadership is crucial in implementing these principles by incorporating AI into organizational strategies that assist clinicians, alleviate administrative burdens, and intentionally reinvest time savings into significant patient interactions. In the absence of policy and leadership alignment, AI may exacerbate efficiency-driven care models, undermining human connection.

### Limitations

While this study provides valuable insights into the factors influencing rehumanization and dehumanization in AI-enabled health care, certain contextual considerations must be acknowledged. Several participants had considerable international experience in advisory, development, research, and educational roles, as well as in clinical practice. Most notably, this experience was gained within European health care systems, with a few also having experience in the US market. This breadth of expertise enriched the perspectives captured in the research, offering viewpoints that extend beyond a solely domestic context. However, it should be noted that the findings are inevitably influenced by the regulatory, organizational, and cultural environment in which the study was conducted. Future research would benefit from the inclusion of a greater number of experts with diverse international market experience and from comparative studies across different health care systems and cultural contexts. Such studies would enhance the generalizability and applicability of the results, ensuring their relevance to a wider range of settings.

### Conclusions

The study’s findings show that AI does not by itself rehumanize or dehumanize health care; rather, its human impact is determined by how it is regulated, applied, and integrated into routine clinical practice. The findings demonstrate that rehumanization is a contingent outcome influenced by organizational tactics, professional attitudes, governmental decisions, and relational dynamics rather than an inevitable consequence of technological advancement by identifying interrelated macro-, meso-, and microlevel elements. Therefore, intentional alignment between technology innovation and humanistic principles is necessary to ensure that AI enhances rather than detracts from patient-centered care. If AI is to work as a catalyst for more compassionate treatment, future health care systems will need to strike a balance between efficiency gains and ongoing investments in empathy, trust, and meaningful clinician-patient connections.

## Supplementary material

10.2196/82774 Multimedia Appendix 1 Semistructured interview guide across macro-, meso-, and microlevel dimensions.

10.2196/82774 Multimedia Appendix 2 Characteristics and artificial intelligence–related experience of interview participants.

10.2196/82774 Multimedia Appendix 3 Macrolevel factors influencing the rehumanizing and dehumanizing potential of artificial intelligence implementation in health care.

10.2196/82774 Multimedia Appendix 4 Mesolevel factors influencing the rehumanizing and dehumanizing potential of artificial intelligence implementation in health care.

10.2196/82774 Multimedia Appendix 5 Microlevel factors influencing the rehumanizing and dehumanizing potential of artificial intelligence implementation in health care.

10.2196/82774 Checklist 1 COREQ checklist.

## Abbreviations

AI: artificial intelligence
COREQ: Consolidated Criteria for Reporting Qualitative Research
RQ: research question
