# Supplementary material for: Artificial Intelligence in Patient-Centered Care and Macro-, Meso-, and Micro-Level Determinants of Rehumanization and Dehumanization: Qualitative Interview Study
Source: J Med Internet Res. 2026 May 27;28:e82774. doi: 10.2196/82774 (PMC13215629; doi:10.2196/82774)
Supplement: Multimedia Appendix 1 [file jmir-v28-e82774-s001.docx]

## **Multimedia Appendix 1:** Semistructured interview guide across macro-, meso-, and micro-level dimensions

**Macro-level questions**

- What are the main trends currently shaping healthcare?
- What business opportunities and new markets could AI create in healthcare?
- To what extent is the national healthcare system and the training of healthcare professionals a determining factor in the implementation of innovative solutions?
- What differences or similarities can be identified between the public vs. private healthcare sectors in the use of AI?
- In your opinion, how could AI be introduced cost-effectively in the public healthcare system, considering budgetary constraints?
- To what extent can AI-based solutions support the potential shortage of healthcare professionals?
- How common is collaboration between different healthcare institutions and external companies when implementing AI solutions?
- What ethical and data-protection risks may arise when using AI-based solutions?
- What steps can be taken to mitigate these risks (e.g., cybersecurity strategies)?
- How might macro-level factors (national policies, regulations, workforce trends, funding structures, and system-wide technological developments) influence the direction of rehumanization or dehumanization in healthcare?
- What opportunities and challenges do you see emerging from these system-level forces?

**Meso-level questions**

**Organizational operations & institutional processes**

- How can AI-based solutions support the development of patient-centered care?
- In what ways can AI improve communication and information flow with patients?
- How might the doctor–patient relationship change with the introduction of AI?
- How can healthcare providers ensure that AI enhances rather than replaces human interaction in patient care?
- To what extent does the risk of dehumanization appear when using AI, and how can this risk be mitigated?
- How can AI-based solutions support the rehumanization of healthcare?

**Decision support & technological integration**

- What are the potential use cases of AI as a decision-support tool in healthcare?
- How can patients be supported in better understanding their diagnosis through AI tools, and how might this affect treatment decisions and outcomes?
- How can AI systems be made reliable and easy to use for healthcare institutions?
- How can AI-based solutions be integrated into existing IT systems? How challenging is integration given the current infrastructure?

**Organizational acceptance & change management**

- How much acceptance or resistance do healthcare workers show toward AI solutions? How can resistance be reduced?
- How do healthcare workers perceive AI-based solutions: as a tool, a peer, or an assistant?
- To what extent is human oversight required when using AI solutions? What should such oversight include?
- Who typically makes decisions on AI-related investments within healthcare institutions?
- Who are the key stakeholders of AI solutions? What is their role, and what challenges are associated with each stakeholder group?
- How can openness to innovation be supported within healthcare institutions? What tools or approaches are used?
- What competencies need to be developed among healthcare workers as AI solutions become more widespread?
- How might meso-level factors (organizational culture, institutional workflows, leadership decisions, and technology adoption practices within universities, hospitals, and companies) shape rehumanization or dehumanization in healthcare?
- What opportunities and challenges arise at the organizational level?

**Micro-level questions**

**Doctor–patient interaction**

- How does AI influence real-time patient–physician interactions?
- How can empathy and human connection be preserved when AI tools are used?
- How can communication with patients be improved through AI on a personal, direct level?

**Patient-side experience**

- How can AI-based solutions support patients in individually understanding their diagnosis?
- How might this influence individual treatment decisions and outcomes?
- What patient-side challenges may arise during the integration of AI solutions? How can these challenges be mitigated?
- What factors influence patient acceptance of AI-based solutions, and how can acceptance be improved?
- How might the use of AI-based solutions affect individual patients’ trust?

**Healthcare workers at the individual level**

- What individual fears or barriers arise among healthcare workers when AI tools are introduced?
- What micro-level training or support do clinicians need to use AI effectively in everyday practice?
- How can AI systems be made easy for individual clinicians to use?
- How might micro-level factors (individual behaviours, personal attitudes, clinician-patient interactions, and day-to-day AI use) contribute to either rehumanization or dehumanization in care delivery?
- What opportunities and challenges do you see at the individual level?
