# Supplementary material for: Artificial Intelligence in Patient-Centered Care and Macro-, Meso-, and Micro-Level Determinants of Rehumanization and Dehumanization: Qualitative Interview Study
Source: J Med Internet Res. 2026 May 27;28:e82774. doi: 10.2196/82774 (PMC13215629; doi:10.2196/82774)
Supplement: Multimedia Appendix 2 [file jmir-v28-e82774-s002.docx]

**Multimedia Appendix 2:** Characteristics and AI-related experience of interview participants

| **ID** | **Title** | **Description** | **Gender** | **Experience with AI** | **International experience** | **Interview duration** |
| --- | --- | --- | --- | --- | --- | --- |
| 1 | Managing Consultant,  PhD Candidate | Health economist and PhD candidate specializing in medicine and health sciences, bringing a strong academic background and a strategic mindset to her role as a Managing Consultant in the Life Sciences sector at a leading management consulting firm. | Woman | Extensive research and consulting experience in the design and implementation of AI-driven healthcare solutions; regular professional and personal use of LLM-based tools. | USA | 46 min. |
| 2 | Assistant Clinical Project Manager,  PhD Candidate | Clinical Research Professional with a PhD in progress and over 5 years of extensive experience across the pharmaceutical industry, clinical research, molecular and microbiology laboratories, and academic research, with a focus on digital healthcare and AI. | Woman | Developer of a self-designed LLM-based AI application for early diagnosis of cervical cancer; regular personal use of LLM-based tools | N/A | 49 min. |
| 3 | Engagement Manager in Management Consulting | Strategic Consultant with over 7 years of international experience at a Big4 company, primarily focused on life sciences projects, strategic partnerships, digitalization initiatives, sustainability efforts, international M&A transactions, and various sell- and buy-side due diligences. | Man | Extensive consulting experience in AI design and implementation projects; regular professional and personal use of LLM-based tools. | CEE region, Germany, Italy | 57 min. |
| 4 | Internal Medicine Specialist, Endocrino-logist | Doctor at a hospital since 2012 and Assistant Professor since 2020. He obtained his general medical degree in 2012 with summa cum laude honors and passed his specialist examinations in internal medicine in 2018 and endocrinology in 2020 with excellent results. | Man | Regular clinical use of an internally developed LLM-based decision-support tool for diagnostic reasoning; personal use of LLM-based tools. | N/A | 45 min. |
| 5 | Head Physician, Rheumato-logist and Physio-therapist | Head Physician at a hospital since 1983. She obtained her general medical degree in 1980 with *summa cum laude* honors and passed her specialist examinations in rheumatology and physiotherapy in 1985 with excellent results. | Woman | No direct professional or personal use of AI-based or LLM-based tools. | Romania | 67 min. |
| 6 | Doctor of General Medicine,  Resident Doctor,  PhD Candidate | Doctor at a hospital since 2023, currently working as a Resident Doctor and PhD candidate at a medical university specializing in medicine and health sciences. He specializes in pediatrics and has a strong interest in the application of artificial intelligence in healthcare. | Man | Regular clinical use of an internally developed LLM-based tool for diagnostic support; personal use of LLM-based tools. | None | 49 min. |
| 7 | Lawyer,  PhD Candidate | Lawyer at an international law firm with over 7 years of experience, particularly in life sciences and pharma-related cases. She is a PhD candidate in Information Technology Law specializing in legal studies, and a member of the Digital Authoritarianism Research Lab. | Woman | Extensive professional experience with AI-related regulatory and governance projects; regular professional and personal use of LLM-based tools. | Austria | 46 min. |
| 8 | Data Science Manager | Leading expert on artificial intelligence and machine learning at a Big4 company, delivering in-house and conference lectures primarily on the practical applications of these technologies. | Man | Extensive consulting experience in the design and implementation of AI and machine learning solutions; regular use of LLM-based tools. | N/A | 47 min. |
| 9 | Associate Professor,  Health Economist,  Data-Driven Healthcare Expert | Associate Professor with over 11 years of experience in health economics and health security, currently working at a Health Services Management Training Centre. He also serves as Vice President of a healthcare-focused Management Association. | Man | Extensive experience in the design, implementation, and evaluation of AI-based healthcare analytics and decision-support systems, including clinical and governmental advisory roles. | N/A | 62 min. |
| 10 | Dentist, CEO | Dentist with a strong commitment to innovation in healthcare. He founded a platform designed to support clinical practice and advance digital health solutions, leveraging AI to integrate technology into medical workflows, enhance efficiency, improve patient outcomes, and foster collaboration among healthcare professionals. | Man | Founder and developer of a proprietary AI-based clinical assistant for physicians; regular professional and personal use of LLM-based tools. | Germany | 52 min. |
| 11 | MedTech Founder, Chief Scientific Officer,  Research Physi-cian, Molecu-lar Pharma-cologist | Cancer researcher and founder of a healthcare AI company, renowned for his work in personalized medicine. His company applies molecular genetics, genomics, and AI to cancer therapy, focusing on developing personalized treatment approaches tailored to individual patients. His work has contributed in a pioneering way to translating molecular medicine research into the practical, personalized treatment of cancer patients. | Man | Founder and lead developer of AI-based precision oncology decision-support solutions integrating molecular and genomic data; regular use of LLM-based tools. | USA, Germany, Slovenia | 50 min. |
| 12 | Senior Manager, eHealth Consultant, Lawyer | Senior Manager at a Big 4 company, overseeing and executing complex public sector and healthcare projects, with expertise in business analysis, business intelligence, IT strategy, data security, and data protection. Brings 6 years of consulting experience, managing teams and clients. | Man | Extensive consulting experience in AI implementation projects in healthcare and public-sector settings; regular use of LLM-based tools. | N/A | 52 min. |
| 13 | International Partner | Partner at a top management consulting firm with over 25 years of experience, specializing in digitalization, IT transformation, artificial intelligence, strategy, process optimization, organizational design, controlling, program management, and cybersecurity. | Man | Extensive consulting experience in AI implementation projects across healthcare and related sectors; regular use of LLM-based tools. | Europe-wide, Middle East, and Africa | 47 min. |
| 14 | Managing Director and Partner | Partner at a top management consulting firm with over 17 years of experience, specializing in advising insurers, health insurers, healthcare institutions, and governments across multiple continents. | Man | Extensive consulting experience in the design and implementation of AI-driven healthcare solutions; regular use of LLM-based tools. | Europe-wide | 51 min. |
| 15 | Company Manager at a Private Clinic | Experienced Customer Relationship Management Lead at a private hospital with a proven track record in the pharmaceuticals industry. Skilled in data analysis, secondary research, communication, strategic planning, and customer relationship management. | Woman | Active involvement in clinical digital transformation and AI integration projects; regular use of LLM-based tools. | N/A | 56 min. |
| 16 | Deputy Director, Health Policy and Communi-cation | Results-oriented healthcare solutions professional with 7+ years of experience leading the full-cycle design and execution of public health programs and strategies. Serves as a member of the Health Emergency Preparedness and Response Authority Advisory Forum. | Man | Extensive advisory experience in AI implementation projects related to public health and healthcare systems; regular use of LLM-based tools. | Singapore, Belgium, UK | 64 min. |
| 17 | Oxylogist, Physio-therapist, Manual Therapist | Emergency medicine specialist, physiotherapy specialist, manual therapist, kinesio tape therapist, medical masseur, and horticultural engineer specialized in medicinal plants. Additionally qualified as a bodybuilding and fitness instructor, and spine trainer, with 25 years of professional experience. | Woman | Regular use of LLM-based tools for clinical brainstorming and reflective practice. | N/A | 52 min. |
| 18 | Senior Physician, Head of Centre of Oncoradio-logy | Internal medicine specialist, clinical oncologist, and clinical pharmacologist with over 35 years of professional experience, certified healthcare manager, and holder of the European Certification in Medical Oncology. Extensive expertise in the diagnosis of malignant diseases and application of modern oncological therapies. | Man | Regular clinical use of LLM-based tools for diagnostic reasoning and case discussion. | None | 55 minutes |
| 19 | Physician,  Head of the Department of Emergency | Certified emergency physician and researcher with expertise in molecular and network sciences, and recent experience in clinical trials. He is the Head of the Department of Emergency Medicine at a university hospital and Associate Professor in Emergency Medicine. | Man | Active involvement in the development and research of AI-based healthcare solutions, including emergency medicine AI triage systems; regular use of LLM-based tools. | Switzerland, UK, USA | 58 min. |
| 20 | Physician,  Anaesthesio-logist | Anaesthesiologist with 10+ years of experience in public hospitals across both the countryside and the capital. Her professional background covers perioperative patient care, intensive therapy, and pain management. During her residency, she also gained international experience in Switzerland. | Woman | Regular professional and personal use of LLM-based tools in clinical practice and decision support. | Switzerland | 45 min. |
