# Supplementary material for: Artificial Intelligence in Patient-Centered Care and Macro-, Meso-, and Micro-Level Determinants of Rehumanization and Dehumanization: Qualitative Interview Study
Source: J Med Internet Res. 2026 May 27;28:e82774. doi: 10.2196/82774 (PMC13215629; doi:10.2196/82774)
Supplement: Multimedia Appendix 3 [file jmir-v28-e82774-s003.docx]

## **Multimedia Appendix 3:** Macro-level factors influencing the rehumanizing and dehumanizing potential of AI implementation in healthcare

| **Factor** | **Conceptual definition** | **Rehumanization mechanisms** | **Dehumanization mechanisms** |
| --- | --- | --- | --- |
| **Regulatory and legal framework** | Refers to the legal, ethical, and institutional norms—such as GDPR or the EU AI Act in the European Union—that govern how AI can be used in healthcare | - Protects patient data and ensures ethical AI use - Builds trust via clear legal frameworks - Safeguards vulnerable groups through oversight | - Overly rigid rules limit flexible AI use - Legal uncertainty delays or distorts implementation - Compliance demands may shift focus from care to bureaucracy |
| **Policy leadership and strategic governance** | Covers political commitment and funding for AI in healthcare through strategies, institutions, and policies | - Value-based agendas align AI with equity and care quality - Strategic coordination enables ethical, scalable transformation - Transparency fosters engagement and societal readiness | - Cost-driven AI use may lead to workforce cuts - Political misuse can hinder patient-centered innovation - Absent vision results in fragmented, exclusionary AI use |
| **Health workforce and labor** | Captures workforce availability, skills, and well-being, including structural issues such as specialist shortages, undercompensated support staff, and digital literacy gaps | - AI can ease admin burdens and cognitive load - Frees up time for empathic patient care - Enhances diagnosis and decisions in low-resource settings | - Replacing (not supporting) clinicians may reduce human connection - De-skilling and marginalization risk clinician disengagement - Algorithmic dominance can constrain judgment and personalized care |
| **Digital infrastructure and data ecosystems** | Covers the technical and organizational foundations for digital health and AI, including IT infrastructure (e.g., EHRs, interoperability), computational capacity, national data repositories, and governance | - Interoperable systems reduce fragmentation and admin burden - Streamlined access to patient information promotes care continuity and strengthens trust - Integrated data enables early, personalized, proactive care | - Siloed information systems create inefficiencies and obstruct patient-centered care - Inconsistent data management undermines the reliability of AI-driven decision support - Inefficiencies and gaps risk errors, duplication, and lost trust |
| **Healthcare system structure and financing logic** | Describes how healthcare system structure and funding—public vs. private, centralized vs. market-based—shape AI adoption. Includes insurance models and incentives that influence access, delivery, and whether AI serves quality, efficiency, or profit | - Public systems can leverage AI for equity and continuity - Preventive-focused insurance encourages patient-centered AI - Data-driven planning supports fair, efficient resource use | - Market incentives may steer AI toward cost-cutting - Private insurance can limit access to privileged groups - Profit-driven models may neglect complex or unprofitable care |
| **Structural accessibility and equity in healthcare provision** | Structural and socio-economic determinants—such as facility location, the balance between public and private provision, insurance coverage, and affordability—shape healthcare access beyond the digital divide | - AI can expand care to underserved areas via remote tools - Public oversight can reduce inequities in AI rollout - Algorithmic triage and resource allocation can improve fairness | - Market-driven AI may disproportionately benefit affluent regions - Uninsured or digitally disconnected populations risk exclusion - Automation may intensify underinvestment in disadvantaged or rural areas |
| **Sociocultural attitudes and health norms** | Captures cultural norms and values shaping healthcare delivery and perception—including views on autonomy, illness, aging, and shared decision-making. Beliefs about individual vs. collective responsibility influence how AI in health is accepted and interpreted | - AI can support culturally sensitive, personalized care - Inclusive design may reduce stigma around illness - Sociotechnical integration may empower patient autonomy - AI may balance power in physician-centered systems | - Standardized AI systems may conflict with diverse cultural and health beliefs - Biases in algorithms can marginalize cultural perspectives - AI may overlook symbolic or spiritual aspects of care - In individualist systems, AI can deepen care commodification |
| **Demographic and generational trends in healthcare demand** | Refers to demographic shifts and generational differences that shape healthcare needs and attitudes toward AI—such as aging populations, digital literacy gaps, and varying expectations of care and autonomy | - AI can support complex care for aging populations - Technology can enable aging in place through remote monitoring and home-based support - Inclusive design promotes late-life autonomy - Digital-savvy generations facilitate participatory and personalized care models | - Systems that ignore cognitive or functional limits risk excluding older adults - Replacing human care with AI risks isolation - Ageist bias can marginalize the elderly - Tech mistrust may reduce care seeking |
