# Supplementary material for: Artificial Intelligence in Patient-Centered Care and Macro-, Meso-, and Micro-Level Determinants of Rehumanization and Dehumanization: Qualitative Interview Study
Source: J Med Internet Res. 2026 May 27;28:e82774. doi: 10.2196/82774 (PMC13215629; doi:10.2196/82774)
Supplement: Multimedia Appendix 4 [file jmir-v28-e82774-s004.docx]

## **Multimedia Appendix 4:** Meso-level factors influencing the rehumanizing and dehumanizing potential of AI implementation in healthcare

| **Factor** | **Conceptual definition** | **Rehumanization mechanisms** | **Dehumanization mechanisms** |
| --- | --- | --- | --- |
| **Institutional vision and strategic integration** | Refers to the extent to which healthcare institutions incorporate AI into their organizational strategies, operational goals, and innovation pathways | - Patient-centered institutions may adopt AI to enhance access and interaction - Strategic planning enables context-driven, burden-reducing AI use - University hospitals and research centers may promote evidence-based, ethically informed AI deployment | - Reactive AI use prioritizes novelty over need - Without strategy, AI use can be ad hoc and unfocused - Misaligned incentives may prioritize efficiency over care quality - Strategic gaps risk redundancy and careless automation |
| **Leadership orientation and change management** | Captures how institutional leaders understand, prioritize, and manage the introduction of AI | - Inclusive leaders frame AI as a care-enhancing tool - Support for pilots fosters learning and ethics - Change management builds shared ownership of AI | - Top-down AI mandates risk resistance or blind compliance - Efficiency-focused leadership may overlook ethics and relationships - Rushed rollout can disrupt professional practice |
| **Organizational culture and professional attitudes** | Encompasses the shared values, norms, and expectations that influence how staff within an institution relate to innovation, hierarchy, and patient care | - Reflective cultures use AI to support, not replace, clinical judgment - Staff-driven innovation aligns AI with professional values - Valuing diverse experiences eases obsolescence fears and affirms human roles | - Fearful environments may resist or misuse AI - AI threatening identity can prompt defensiveness - Tokenism risks reducing AI to symbolism, harming care quality |
| **Information technology infrastructure and integration** | Refers to the state and interoperability of digital systems within healthcare institutions, including the presence of legacy software, device compatibility, data bandwidth, and connection to national platforms | - Modular AI integration supports clinical workflows and decisions - Streamlined systems improve information access and patient care - Secure cloud systems broaden access without straining local resources | - Legacy systems fragment data and care - Poor integration burdens clinicians with disconnected tools - Tech overload can shift focus from patients to screens |
| **Training, education, and interdisciplinary capacity** | Refers to the institutional and academic structures that facilitate AI-related education in both healthcare systems and higher education | - Interdisciplinary training fosters ethics and cross-sector understanding - Critical AI literacy empowers future professionals to challenge tech determinism - Ongoing, inclusive training builds responsible, relational AI use - Co-created education promotes shared ownership and governance of AI tools | - Inaccessible training can disempower staff or fuel resistance - Technocentric education may ignore care’s relational side - Lack of critical AI engagement fosters passive use - Educational gaps can deepen systemic exclusion in healthcare across roles and generations |
