# Supplementary material for: Artificial Intelligence in Patient-Centered Care and Macro-, Meso-, and Micro-Level Determinants of Rehumanization and Dehumanization: Qualitative Interview Study
Source: J Med Internet Res. 2026 May 27;28:e82774. doi: 10.2196/82774 (PMC13215629; doi:10.2196/82774)
Supplement: Multimedia Appendix 5 [file jmir-v28-e82774-s005.docx]

## **Multimedia Appendix 5:** Micro-level factors influencing the rehumanizing and dehumanizing potential of AI implementation in healthcare

| **Factor** | **Conceptual definition** | **Rehumanization mechanisms** | **Dehumanization mechanisms** |
| --- | --- | --- | --- |
| **Clinician trust in AI systems** | Refers to healthcare professionals’ confidence in the reliability, clinical relevance, and ethical soundness of AI tools | - Clinician involvement boosts AI acceptance - Transparency and explainability build confidence - AI supporting (not replacing) judgment strengthens trust | - Lack of transparency or explainability may undermine confidence and autonomy - AI seen as replacing expertise triggers resistance - Unconsulted use of AI risks identity threat and disengagement |
| **Patient trust and receptivity** | Refers to patients’ willingness to accept AI-supported healthcare interventions, shaped | - Clear, empathetic AI communication supports consent and agency - Familiarity with AI tools fosters openness and normalization - Accuracy and personalization boost legitimacy and engagement | - Poor transparency and impersonal AI lower care quality and alienate patients - Patients may expect more from AI, reducing error tolerance - Digital exclusion undermines confidence and trust |
| **Generational and digital literacy differences** | Captures interindividual differences in digital competence and openness to innovation, often associated with generational identity | - Younger, tech-savvy users drive cultural adaptation - Targeted training reduces digital exclusion - Intergenerational learning promotes inclusion and understanding | - Limited digital skills among older users can lead to exclusion or dependence - Tech unfamiliarity raises anxiety and lowers engagement - Digital access gaps can deepen health and social inequities |
| **Emotional and psychological readiness** | Refers to the emotional and cognitive state of individuals—such as burnout, stress, or openness to change—that affects their engagement with AI | - AI easing admin tasks can reduce burnout and improve work-life balance - Presenting AI as supportive boosts readiness - Positive AI experiences enhance motivation and purpose | - Burnout hampers learning and adaptation - Complex or controlling AI increases stress - Psychological disengagement can breed resistance or apathy toward innovation |
| **Interpersonal empathy and relational capacity** | Describes the clinician’s ability to communicate empathetically and maintain meaningful interpersonal relationships with patients, especially in AI-mediated contexts | - AI time savings enable more patient-centered care - Training in empathy and communication enhances human connection in tech-driven care - Clinician oversight preserves trust and accountability | - Delegating emotional or communicative tasks to AI reduce empathy - Overreliance on data may marginalize patients’ narratives - Less face-to-face time risks weakening the therapeutic bond |
| **AI literacy and data orientation** | Refers to individuals’ ability to understand, critically evaluate, and appropriately use AI tools and data-driven outputs in healthcare contexts | - AI literacy enables critical, effective engagement with AI - Data skills promote informed, personalized care - Institutional training builds confident, autonomous use | - Limited technical understanding may lead to misuse or uncritical use of AI - Lack of data skills may cause mistrust of AI insights - Uncertainty about AI can undermine confidence and decisions |
| **Doctor–patient interaction dynamics** | Encompasses the communicative, ethical, and power dynamics between clinicians and patients | - AI improves shared decisions via accessible information - Transparent AI use empowers collaboration - Shifting knowledge authority fosters respect and reduces hierarchy | - Algorithmic authority may concentrate power and obscure accountability - Excluding patients from AI decisions harms trust and autonomy - Automated processes risk depersonalizing care |
