# Supplementary material for: Artificial Intelligence in Patient-Centered Care and Macro-, Meso-, and Micro-Level Determinants of Rehumanization and Dehumanization: Qualitative Interview Study
Source: J Med Internet Res. 2026 May 27;28:e82774. doi: 10.2196/82774 (PMC13215629; doi:10.2196/82774)
Supplement: Checklist 1 [file jmir-v28-e82774-s006.docx]

# COREQ (Consolidated Criteria for Reporting Qualitative Studies): 32-Item Checklist

##

| **No.** | **Item** | **Guide question** | **Response** |
| --- | --- | --- | --- |
| **Domain 1: Research team and reflexivity** | | | |
| Personal characteristics | | | |
| 1. | Interviewer/facilitator | Which author/s conducted the interview or focus group? | Dóra Horváth and Noémi Szilvia Lőrincz carried out the interviews. |
| 2. | Credentials | What were the researcher’s credentials? *E.g. PhD, MD* | Dóra Horváth, PhD  Noémi Szilvia Lőrincz, PhD |
| 3. | Occupation | What was their occupation at the time of the study? | Dóra Horváth, PhD – Assistant Professor at the Corvinus University of Budapest  Noémi Szilvia Lőrincz, PhD – Assistant Lecturer at the Corvinus University of Budapest |
| 4. | Gender | Was the researcher male or female? | 2 female researchers |
| 5. | Experience and training | What experience or training did the researcher have? | Both researchers have years of experience in academic research. |
| Relationship with participants | | | |
| 6. | Relationship established | Was a relationship established prior to study commencement? | There was no relationship established with the participants prior to the study commencement. |
| 7. | Participant knowledge of the interviewer | What did the participants know about the researcher? *e.g. personal goals, reasons for doing the research* | The participants knew that the interviewers were part of a scientific research group at Corvinus University of Budapest, with a common objective of improving knowledge about how artificial intelligence can influence patient-centered care. |
| 8. | Interviewer characteristics | What characteristics were reported about the interviewer/facilitator? *e.g. Bias, assumptions, reasons and interests in the research topic* | The interviewers were researchers with academic expertise and interest in artificial intelligence and healthcare. Their motivation was to explore its impact on patient-centered care. No specific biases or assumptions were explicitly reported. |

| **Domain 2: Study design** | | | |
| --- | --- | --- | --- |
| Theoretical framework | | | |
| 9. | Methodological orientation and Theory | What methodological orientation was stated to underpin the study? *e.g. grounded theory, discourse analysis, ethnography, phenomenology, content analysis* | Grounded theory (page 6) |
| Participant selection | | | |
| 10. | Sampling | How were participants selected? *e.g. purposive, convenience, consecutive, snowball* | To identify appropriate participants for the research, purposive sampling was employed, which is a widely used technique in qualitative research. (page 7) |
| 11. | Method of approach | How were participants approached? *e.g. face-to-face, telephone, mail, email* | E-mail approach (page 7) |
| 12. | Sample size | How many participants were in the study? | 20 participants (page 7) |
| 13. | Non-participation | How many people refused to participate or dropped out? Reasons? | 6 participants. The main reason for not participating was that they did not have time or had no interest. |
| Setting | | | |
| 14. | Setting of data collection | Where was the data collected? *e.g. home, clinic, workplace* | At participants’ workplaces or via secure online platforms, depending on their preferences. (page 7) |
| 15. | Presence of non-participants | Was anyone else present besides the participants and researchers? | No, the interviewers and the participants were alone during the interview. |
| 16. | Description of sample | What are the important characteristics of the sample? *e.g. demographic data, date* | Please see Appendix 2: Characteristics and AI-Related experience of interview participants |
| Data collection | | | |
| 17. | Interview guide | Were questions, prompts, guides provided by the authors? Was it pilot tested? | The authors provided an interview guideline. |
| 18. | Repeat interviews | Were repeat interviews carried out? If yes, how many? | No |
| 19. | Audio/visual recording | Did the research use audio or visual recording to collect the data? | All interviews were audio-recorded with the informed consent of the participants, who were thoroughly briefed on the purpose of the research and the procedures for data handling. (page 8) |
| 20. | Field notes | Were field notes made during and/or after the interview or focus group? | Yes |
| 21. | Duration | What was the duration of the interviews or focus group? | Interview duration ranged from 45 to 67 minutes. (page 10) |
| 22. | Data saturation | Was data saturation discussed? | Yes (page 6) |
| 23. | Transcripts returned | Were transcripts returned to participants for comment and/or correction? | No |

| **Domain 3: Analysis and findings** | | | |
| --- | --- | --- | --- |
| Data analysis | | | |
| 24. | Number of data coders | How many data coders coded the data? | All interviews were conducted jointly by both authors, who also collaborated closely throughout the data analysis process, including coding, theme development, and interpretation. (page 7) |
| 25. | Description of the coding tree | Did authors provide a description of the coding tree? | No, the authors did not provide an explicit coding tree. However, the coding structure was described through first-order concepts, second-order themes, and aggregate dimensions following the Gioia methodology. (page 9) |
| 26. | Derivation of themes | Were themes identified in advance or derived from the data? | Themes were derived from the data. |
| 27. | Software | What software, if applicable, was used to manage the data? | Audio-recorded interviews were coded using NVivo. |
| 28. | Participant checking | Did participants provide feedback on the findings? | We will offer it when they are published. |
| Reporting | | | |
| 29. | Quotations presented | Were participant quotations presented to illustrate the themes / findings? Was each quotation identified? *e.g. participant number* | Yes |
| 30. | Data and findings consistent | Was there consistency between the data presented and the findings? | Yes |
| 31. | Clarity of major themes | Were major themes clearly presented in the findings? | Yes |
| 32. | Clarity of minor themes | Is there a description of diverse cases or discussion of minor themes? | Yes |
